# Tuning the Air Stability of N‐Type Semiconductors via Poly(2‐vinylpyridine): The Importance of Humidity and Molecular Weight

**DOI:** 10.1002/smsc.202500452

**Published:** 2025-10-23

**Authors:** Laura E. Dickson, Vittoria‐Ann DiPalo, Trevor Plint, Kannan Udaya Mohanan, Joseph G. Manion, Chang‐Hyun Kim, Benoît H. Lessard

**Affiliations:** ^1^ Department of Chemical and Biological Engineering University of Ottawa 161 Louis Pasteur Ottawa Ontario K1N 6N5 Canada; ^2^ School of Electrical Engineering and Computer Science University of Ottawa 800 King Edward Ave. Ottawa Ontario K1N 6N5 Canada

**Keywords:** air stability, N‐type semiconductor stability, organic thin‐film transistors, polymer blending

## Abstract

The environmental instability of n‐type semiconducting polymers remains a limitation for organic thin‐film transistors (OTFTs), as oxygen diffusion and oxidation reduces device performance. Herein, a simple stabilization strategy using poly(2‐vinylpyridine) (P2VP), a synthetically accessible, hygroscopic, insulating polymer, is shown. Building on earlier work showing short‐term stabilization with this insulating additive, the molecular weight of P2VP is systematically varied and it is demonstrated that higher molecular weight chains form larger domains that reduce oxygen access to the crystalline regions of the benchmark n‐type polymer P(NDI2OD‐T2). Structural characterization reveals that P2VP domains absorb atmospheric moisture, which both decreases the free volume available for oxygen penetration and partitions oxygen away from semiconductor crystallites. As such, devices containing P2VP exhibit enhanced stability over seven days and can be regenerated by mild heating, whereas neat P(NDI2OD‐T2) devices remain degraded. These findings provide mechanistic insight into how insulating polymer blends mediate oxygen–water interactions and highlight polymer blending as a scalable strategy for improving the operational stability of n‐type OTFTs.

## Graphical abstract

Blending the n‐type polymer P(NDI2OD‐T2) with hygroscopic poly(2‐vinylpyridine) (P2VP) improves device stability by limiting oxygen diffusion. Higher molecular weight P2VP forms larger domains that absorb moisture, reduce free volume for oxygen, and partition oxygen away from semiconductor crystallites. This scalable strategy enhances operational stability of organic thin‐film transistors and enables device regeneration by mild heating.

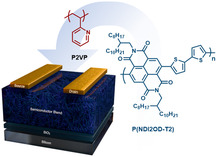

## 1 Introduction

Organic thin‐film transistors (OTFTs) are essential components for flexible, large‐area, low‐cost electronics.^[^ ^1^, ^2^, ^3^ ^]^ However, to fully realize the potential of OTFTs, integrated complimentary logic circuits are required, which demand balanced performance from both electron‐transporting (n‐type) and hole transporting (p‐type) organic semiconductors.^[^ ^4^, ^5^ ^]^ Significant advances have been made to develop p‐type materials, however, limitations still exist for n‐type semiconductors due to their sensitivity under ambient conditions.^[^ ^6^ ^]^ In particular, n‐type OTFTs, such as those based on naphthalene diimide derivatives (e.g., P(NDI2OD‐T2)), suffer from poor air stability, threshold voltage (*V_t_*) drift, and reduced operational lifetimes compared to their p‐type counterparts.^[^ ^7^ ^]^ This is a direct result of their lowest unoccupied molecular orbital level aligning close to the redox potentials of oxygen and water, making them prone to oxidative degradation and electron trapping.^[^ ^5^, ^6^, ^8^, ^9^, ^10^, ^11^, ^12^, ^13^, ^14^ ^]^ This instability not only reduces device performance but also accelerates local heating, unstable transport, and eventual device failure.^[^ ^5^ ^]^

A wide range of strategies have been explored to address these operational instabilities, including molecular design, device engineering, and encapsulation.^[^ ^15^, ^16^, ^17^, ^18^, ^19^, ^20^, ^21^ ^]^ While effective to some degree, methods like encapsulation increase fabrication complexity, add costs, and do not address intrinsic degradation pathways that originate within the semiconductor bulk or at critical interfaces.^[^ ^15^, ^16^, ^17^, ^18^, ^19^, ^20^, ^21^ ^]^ Consequently, strategies that enhance stability while maintaining electronic performance and processing simplicity remain a topic of interest.^[^ ^5^ ^]^

In recent years, blending n‐type semiconducting polymers with insulating polymers has emerged as a promising strategy to improve device operational stability without severely compromising charge transport.^[^ ^4^, ^5^, ^7^, ^8^, ^22^, ^23^, ^24^, ^25^, ^26^, ^27^, ^28^, ^29^ ^]^ Insulating additives can reduce environmental degradation, mechanically stabilize the film, and tune solution processibility, while the n‐type component preserves conduction pathways.^[^ ^5^, ^30^, ^31^ ^]^ These properties are highly dependent on the molecular interactions between blend components and the resulting structural arrangements of polymer chains within the blend.^[^ ^32^ ^]^ The interplay between microstructure and electrical properties in conjugated polymers is far from being perfectly understood, however, it has been found that the unifying requirement for high carrier mobility is the presence of interconnected aggregates, even if they are small and disordered.^[^ ^33^ ^]^ Studies have indicated that insulating polymers generally do not penetrate the crystalline semiconductor, instead dispersing within amorphous regions.^[^ ^5^, ^6^, ^8^ ^]^ As a result, the insulating fraction can influence blend morphology, heat dissipation, and modulate electronic pathways in n‐type devices.^[^ ^5^, ^22^ ^]^

We have previously demonstrated that exposing P(NDI2OD‐T2) to amine‐containing additives can alter operational stability when tested from nitrogen to air, with pyridine proving particularly promising, however, device improvement was short lived due to pyridine's volatility.^[^ ^8^ ^]^ This effect was further explored by blending this n‐type semiconductor with poly(2‐vinylpyridine) (P2VP), a pyridine functionalized polymer.^[^ ^6^ ^]^ We found that even at 0.1 wt% loading, P2VP stabilized device performance, while higher concentrations (10–50 wt%) extended stability over a week. Control experiments with structurally similar polystyrene confirm this effect was specific to blends containing pyridine functional groups.

**Figure 1:**
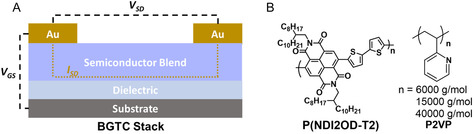
A) Schematic of the BGTC OTFT architecture used to test P(NDI2OD‐T2)/P2VP blends. B) Chemical structures of the n‐type semiconductor P(NDI2OD‐T2) and stabilizing polymer P2VP, showing the range of molecular weights employed in this study.

Here, we extend this work to investigate the mechanisms underlying the stabilization by studying P(NDI2OD‐T2)/P2VP blends as a function of P2VP molecular weight. Specifically, we show how P2VP molecular weight influences film morphology and device performance in bottom‐gate, top‐contact (BGTC) OTFTs (**Figure** **1**). Moreover, we explore the role of atmospheric moisture, and demonstrate that P2VP‐containing devices can be regenerated through a mild thermal treatment, unlike neat P(NDI2OD‐T2), as a result of its hygroscopic properties.^[^ ^34^, ^35^, ^36^, ^37^, ^38^, ^39^, ^40^ ^]^ Finally, we propose that oxidative degradation of P(NDI2OD‐T2) is suppressed by P2VP water uptake, where the absorbed water displaces and partitions oxygen from the semiconductor matrix. In the absence of P2VP, oxygen can readily oxidize P(NDI2OD‐T2), leading to rapid device failure.

This mechanistic insight highlights the importance of polymer–polymer interactions in stabilizing air‐sensitive n‐type OTFTs. Beyond performance metrics, our results establish a framework for designing stable n‐type polymer blend devices and emphasize the role of environmental factors on blend morphology.

## 2 Results and Discussion

### 2.1 Effect of P2VP Molecular Weight

**Figure 2:**
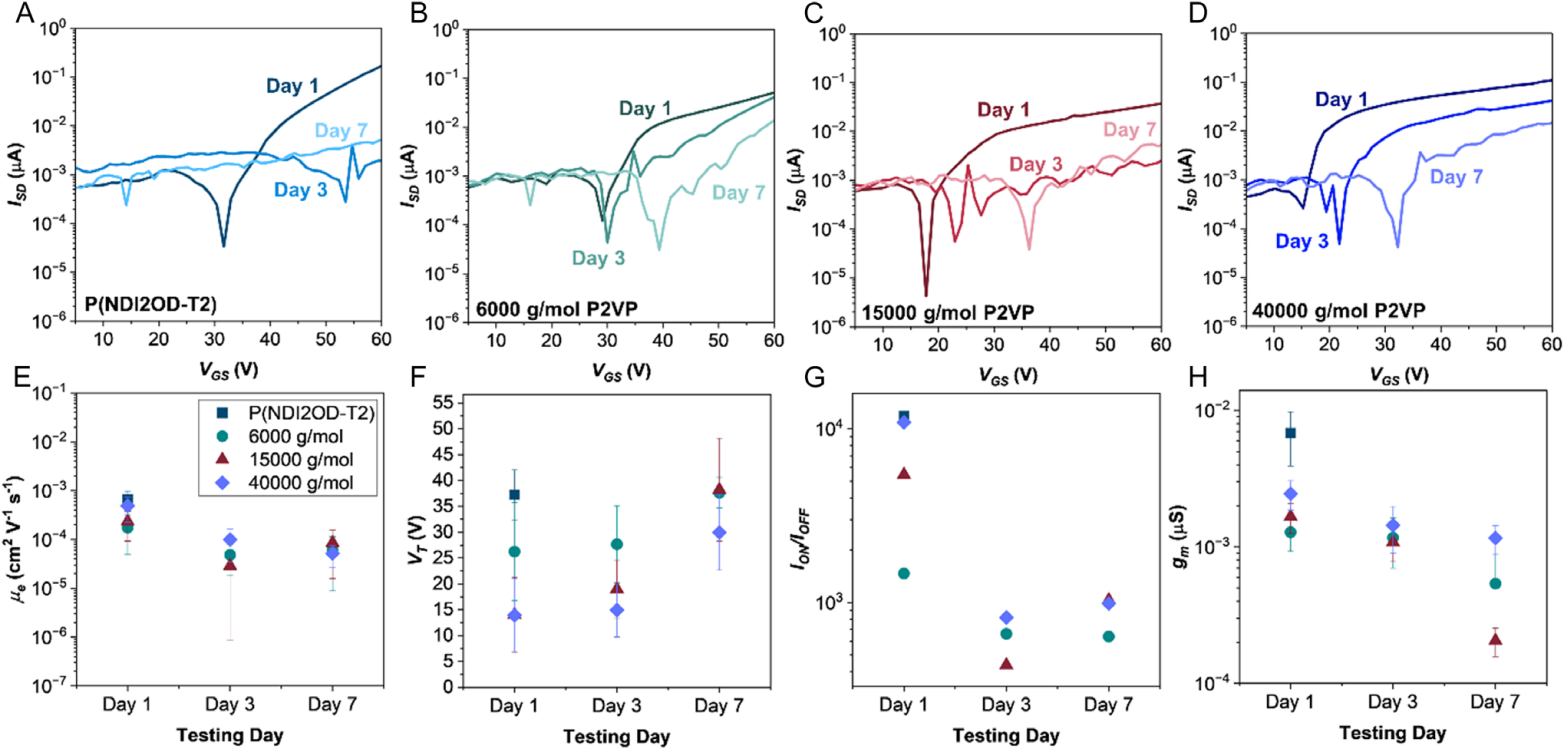
Representative transfer curves for OTFT devices fabricated with A) neat P(NDI2OD‐T2) and 50/50 v/v blends with P2VP of molecular weight (Mn) of B) 6000 g mol^−1^, C) 15000 g mol^−1^, and D) 40000 g mol^−1^. Devices are characterized on fabrication day (day 1), and after 3 and 7 days under ambient conditions. Average performance of 40 devices with standard error: E) electron mobility (*μ*_*e*_), F) threshold voltage (*V*_*T*_ *)*, G) *I*_ON/_ *I*_OFF_ ratio, and H) transconductance (*g*_*m*_).

BGTC OTFTs were fabricated using neat P(NDI2OD‐T2) or 50/50 v/v blends of P(NDI2OD‐T2) with P2VP of varying molecular weights. P2VP was synthesized by nitroxide‐mediated polymerization (NMP), a reversible‐deactivation radical polymerization technique that ensures predictable molecular weight and dispersity (Table S1, Supporting Information).^[^ ^41^ ^]^ Representative transfer curves for neat and blended films, measured over seven days, are shown in **Figure** **2**A–D with blends containing P2VP number average molecular weight (Mn) of 6000, 15 000, and 40 000 g mol^−1^. The extracted electron mobility (*μ*_e_), threshold voltage (*V*_T_ *), I*_ON*/*_ *I*_OFF_ ratio, and transconductance (*g*_m_), are summarized in Figure 2E–H, with sample output curves found in Figure S4, Supporting Information. Measurements were performed on the day of fabrication (day 1), and after 3 (day 3) and 7 days (day 7) of ambient exposure. Notably, while the *μ*_e_ *’*s reported are less than state‐of‐the‐art P(NDI2OD‐T2) devices, they are within the same order of magnitude to reports of spin‐coated films on SiO_2_.^[^ ^6^, ^8^, ^42^, ^43^ ^]^

On day 1, neat P(NDI2OD‐T2) exhibited the highest *g*_m_ (6.84 × 10^−3^ μS), which is consistent with its superior *I*_ON_ */I*_OFF_ ratio and *μ*_e_. Blending with P2VP reduces initial mobility, likely due to the disruption of long‐range order of P(NDI2OD‐T2).^[^ ^28^ ^]^ However, *V*_T_ is significantly reduced for P2VP blends, which is likely a result of the insulator polymer diluting trap states and reducing the potential required for device operation.^[^ ^5^ ^]^ Moreover, a clear molecular‐weight dependence was observed, whereby increasing P2VP Mn led to higher *μ*_e_, *I*_ON_ */I*_OFF_, *g*_m_, and a reduced *V*_T_.

Long‐term stability was markedly improved for P2VP‐containing blends. While neat P(NDI2OD‐T2) films ceased to function after day 1, blends remained operational over 7 days, independent of P2VP Mn. Among the blends, Mn = 40 000 g mol^−1^ yielded the highest stability, which is illustrated by the smallest change in *g*_m_ (7.45 × 10^−4^ μS) compared to 1.29 × 10 and 1.46 × 10^−3^ μS for 6000 and 15 000 g mol^−1^, respectively. These results highlight the role of P2VP molecular weight in promoting long‐term device performance under ambient conditions.

**Figure 3:**
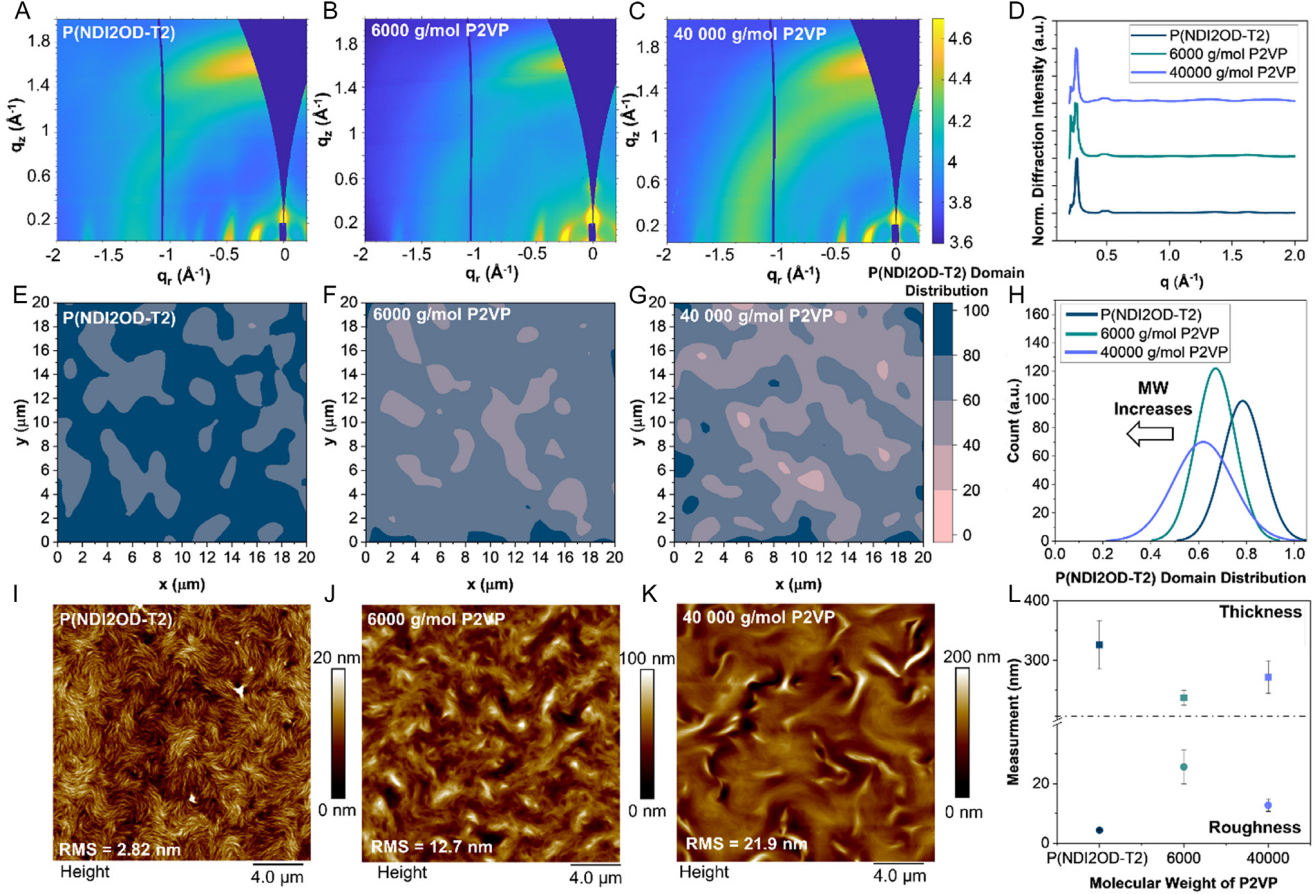
GIWAXS scattering patterns for A) neat P(NDI2OD‐T2) and 50/50 v/v ratio of P2VP with a Mn of B) 6000 g mol^−1^ and C) 40 000 g mol^−1^, and D) the corresponding linecuts. Relative P(NDI2OD‐T2) domain distribution Raman maps for E) neat P(NDI2OD‐T2) and 50/50 blends with a Mn of F) 6000 g mol^−1^ and G) 40 000 g mol^−1^. All three maps share the scale bar shown in (G). H) Histogram of P(NDI2OD‐T2) domain sizes. 20 × 20 μm AFM scans of I) neat P(NDI2OD‐T2), and 50/50 blends of J) 6000 g mol^−1^ and K) 40 000 g mol^−1^ P2VP. L) Average film thickness and surface roughness extracted from six profilometry measurements.

The neat and blended films were then characterized by X‐ray diffraction (Figure S5, Supporting Information). No notable differences in crystallinity were observed, indicating that the intermolecular packing of P(NDI2OD‐T2) is not affected by the molecular weight of P2VP. To further probe the microstructure, synchrotron‐based grazing incident wide angle X‐rays scattering (GIWAXS) was performed on the same films. Corresponding 2D scattering (**Figure** **3**A–C) and line cuts (Figure 3D) confirm that the crystalline domain structure of P(NDI2OD‐T2) remains essentially unchanged irrespective of the P2VP molecular weight. In all cases, the interplanar spacing of the (100) lamellar peak was consistent at *d*_(100)_ = 22.65 Å (q ≈ 0.278 Å^−1^). This observation corroborates our previous findings that P2VP does not penetrate or disrupt the P(NDI2OD‐T2) crystalline domains, but rather segregates into amorphous regions of the blends.^[^ ^5^, ^6^ ^]^

In addition to the structural analysis of crystalline domains, film composition was examined by Raman spectroscopy. Raman mapping is well suited to the polymer blend systems as it is sensitive to variations in local molecular ordering and composition, and is widely reported to effectively visualize phase separation.^[^ ^44^, ^45^, ^46^, ^47^ ^]^ Raman compositional mapping has also been used to probe domain purity and molecular ordering in conjugated/insulating polymer systems by normalized Raman contrast.^[^ ^48^ ^]^ For this system, Raman compositional mapping was performed by plotting the normalized intensity of the P(NDI2OD‐T2) C—C stretching mode at 1610 cm^−1^ for each micron square pixel on the Raman map.^[^ ^49^, ^50^ ^]^ This approach provides spatially resolved information on the distribution of P(NDI2OD‐T2) within the P2VP matrix and enables assessment of how polymer molecular weight influences blend homogeneity.

Raman maps (Figure 3E–G) reveal distinct domains for P(NDI2OD‐T2) and P2VP‐rich regions within the thin films. Notably, even at a constant volume ratio, the P2VP domains become denser and more frequent as the molecular weight increases. This suggests that low‐Mn P2VP chains more readily diffuse or disperse into the amorphous P(NDI2OD‐T2) regions, whereas higher‐Mn form their own distinct domains. This is consistent with prior reports demonstrating Mn‐dependent domain formation in polymer blends.^[^ ^28^, ^51^ ^]^

Qualitative analysis of the Raman maps as compositional histograms further supports this observation. As P2VP Mn increases from 6000 to 40 000 g mol^−1^, P(NDI2OD‐T2) exhibits a broader compositional distributions and lower average density (Figure 3H). For Mn = 6000 g mol^−1^, the average pixel contains ≈80% P(NDI2OD‐T2) with a range between 50% and 100%, whereas for Mn = 40 000 g mol^−1^, the average decreases to ≈55% ranging from 30% to 90%. This indicates that higher‐Mn of the P2VP produces purer and well‐defined P2VP domains, though P(NDI2OD‐T2) remains intermixed in all cases. These results are consistent with our previous report using P2VP Mn = 25 000 g mol^−1^, which showed no pure P2VP domains by scanning transmission X‐ray microscopy.^[^ ^6^ ^]^

Atomic force microscopy (AFM) further corroborates these results (Figure 3I–K). The neat P(NDI2OD‐T2) films exhibit swirling, fiber‐like structures that are reduced upon blending with P2VP, which is also reflected in increased surface roughness and height variations (Figure 3L). Furthermore, the addition of P2VP promotes nanoscale polymer aggregation, forming ring‐like structures that become larger and more numerous as P2VP Mn increases (Figure S6, Supporting Information).^[^ ^51^, ^52^ ^]^ The Raman and AFM results suggest that the observed variation in OTFT stability as a function of P2VP molecular weight are likely a result of corresponding changes in nano‐ and microscale film morphology.^[^ ^28^ ^]^

### 2.2 Effect of Humidity on Device Stability

**Figure 4:**
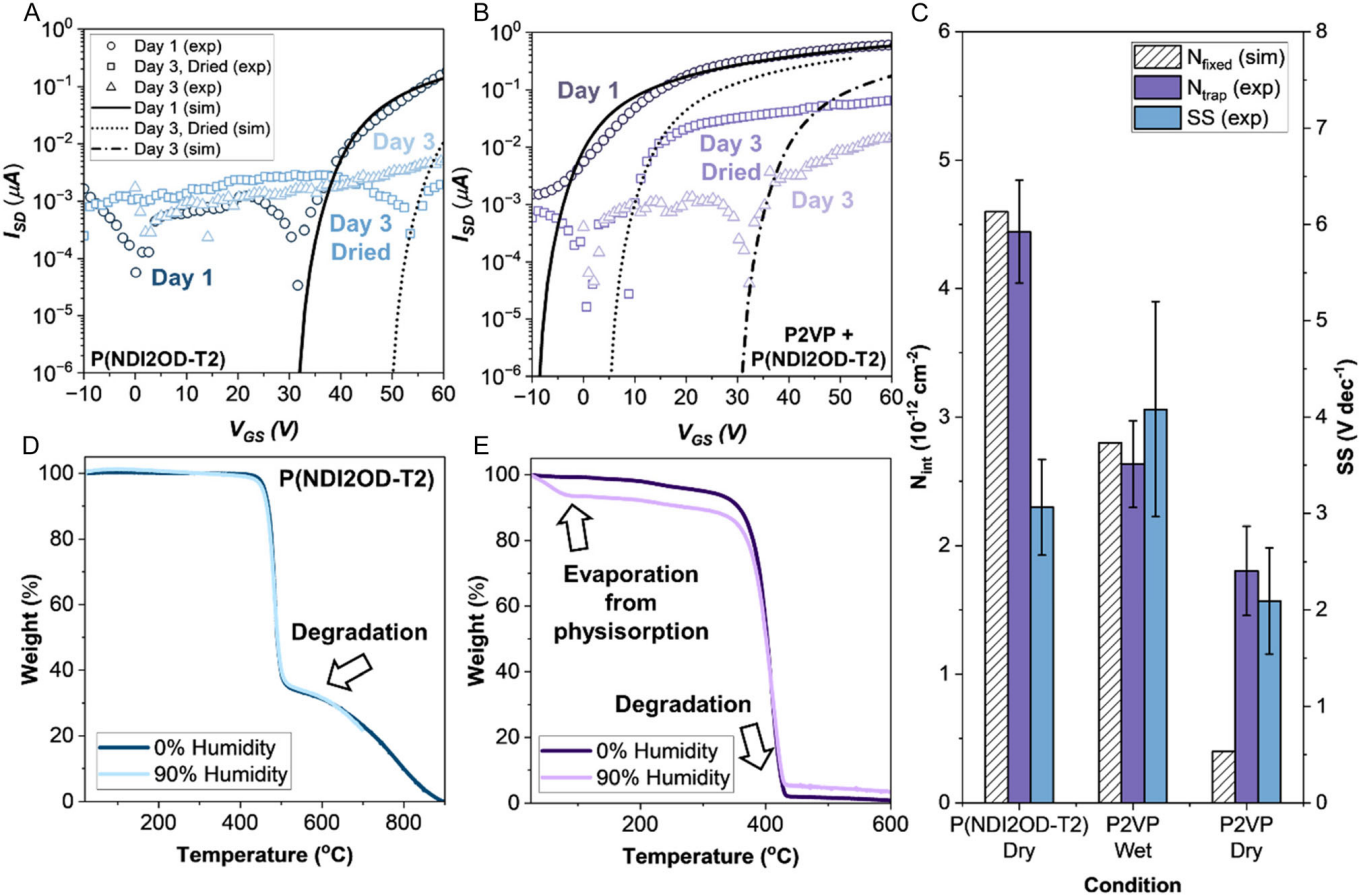
Transfer characteristics and modeling of OTFT devices measured on day 1 and day 3 for A) neat P(NDI2OD‐T2) and B) P2VP/P(NDI2OD‐T2) 20/80 v/v blend. Day 3 devices are shown before (triangle) and after (square) mild thermal treatment (1 h at 50 °C in vacuum). C) Fixed interface charge density (*N*_int,fixed_), interface trap density (*N*_int,trap_), and SS for 40 devices, with error bars representing the standard error. TGA analysis of D) P(NDI2OD‐T2) and E) P2VP after drying (1 h at 50 °C in vacuum) or exposure to 90% RH (1 h).

Initial testing on day 3 revealed that even blended OTFT devices exhibited reduced performance under ambient conditions (**Figure** **4**A,B), in contrast to our previous work that demonstrated stability up to one week.^[^ ^6^ ^]^ This discrepancy is attributed to environmental conditions, where the prior study was conducted during winter (relative humidity (RH) ≈20%) while the current experiments were performed in summer (RH ≈50%).

To assess the role of moisture, devices were regenerated by drying in a vacuum oven. Heating the samples to 50 °C for an hour led to significant recovery in performance of the blended films (Figure 4B), while neat P(NDI2OD‐T2) showed little recovery (Figure 4A). This phenomena is consistent with prior reports that show how the diffusion of water molecules within OTFTs is reversible.^[^ ^53^ ^]^ Unlike previous studies, the absorption of water appears to occur primarily within the stabilizing P2VP polymer rather than the semiconductor itself. This observation suggests that P2VP facilitates recovery of the air‐sensitive P(NDI2OD‐T2) by acting as a moisture reservoir, which can be reversed upon thermal treatment.

To quantify the influence of moisture on device performance, device‐level modeling of the transfer characteristics for P(NDI2OD‐T2) and P2VP‐containing devices was performed on day 1 and day 3, before and after thermal drying (Figure 4A,B). Both interface trap density (*N*_int,trap_) and the fixed interface charge density (*N*_int,fixed_) contribute to pronounced *V*_T_ shifts, arising from the formation of an electric field at the dielectric/semiconductor interface from trapped charges.^[^ ^53^, ^54^, ^55^, ^56^ ^]^ However, the influence of each of these parameters on the transfer characteristics varies: while interface traps modulate the shape and position of the transfer curve, fixed interface charge leads only to a lateral shift without altering curve shape.^[^ ^54^, ^55^, ^56^ ^]^

As shown in Figure 4C, both *N*_int,trap_ and *N*_int,charge_ increases in P2VP‐containing devices following water absorption, with a subsequent decrease upon heating. This reduction correlates to a downshift in *V*_T_, suggesting that desorption of moisture mitigates the formation of interfacial trap sites. Furthermore, analysis of the subthreshold swing (*SS*) reveals an increase in *SS* for devices in the “wet” state, confirming the formation of interfacial trap sites at the critical interface. These findings suggest that water absorbed by P2VP increases interface traps, thereby hindering charge transport and increasing *V*_T_. This is consistent with prior reports identifying water as a major factor in the operational and environmental degradation of OTFTs.^[^ ^13^, ^57^, ^58^ ^]^ In contrast, neat P(NDI2OD‐T2) devices exhibit minimal variation in performance upon heating, suggesting that the moisture‐induced effects are likely associated with the hydrophilic properties of P2VP. Additional extracted electrical parameters are provided in Table S4 and S5, Supporting Information.

Thermogravimetric analysis (TGA) was performed on dried and humidified samples of P(NDI2OD‐T2) and P2VP. Humidified samples were exposed to RH = 90% for an hour in a Linkam stage with controlled humidity. The results (Figure 4D,E **)** indicate that P(NDI2OD‐T2) degrades around 500 °C but shows no physical or chemical water uptake. In contrast, P2VP exhibits initial mass loss at ≈60–100 °C, followed by a secondary loss at ≈420 °C. The first peak is attributed to the physical desorption of water from P2VP, consistent with prior reports that show this occurs below 150 °C.^[^ ^20^, ^21^ ^]^ Meanwhile, chemisorbed water is typically observed above 200 °C, indicating that water is physically binding to the P2VP domains.^[^ ^19^, ^20^ ^]^ The hygroscopic behavior of P2VP has been widely reported and is attributed to the pendant pyridine moieties, whereby the nitrogen atoms readily hydrogen bond to water molecules and become protonated, making the polymer strongly water‐affinitive.^[^ ^34^, ^35^, ^36^, ^37^, ^38^, ^39^, ^40^ ^]^ Therefore, these results suggest that neat P(NDI2OD‐T2) does not absorb water, which may explain why thermal treatment was not able restore device performance. When P2VP is present, physical absorption/desorption of water hinders charge transport, increasing the bias required for operation. However, once the water has been removed, recovery of device performance can be achieved.

**Figure 5:**
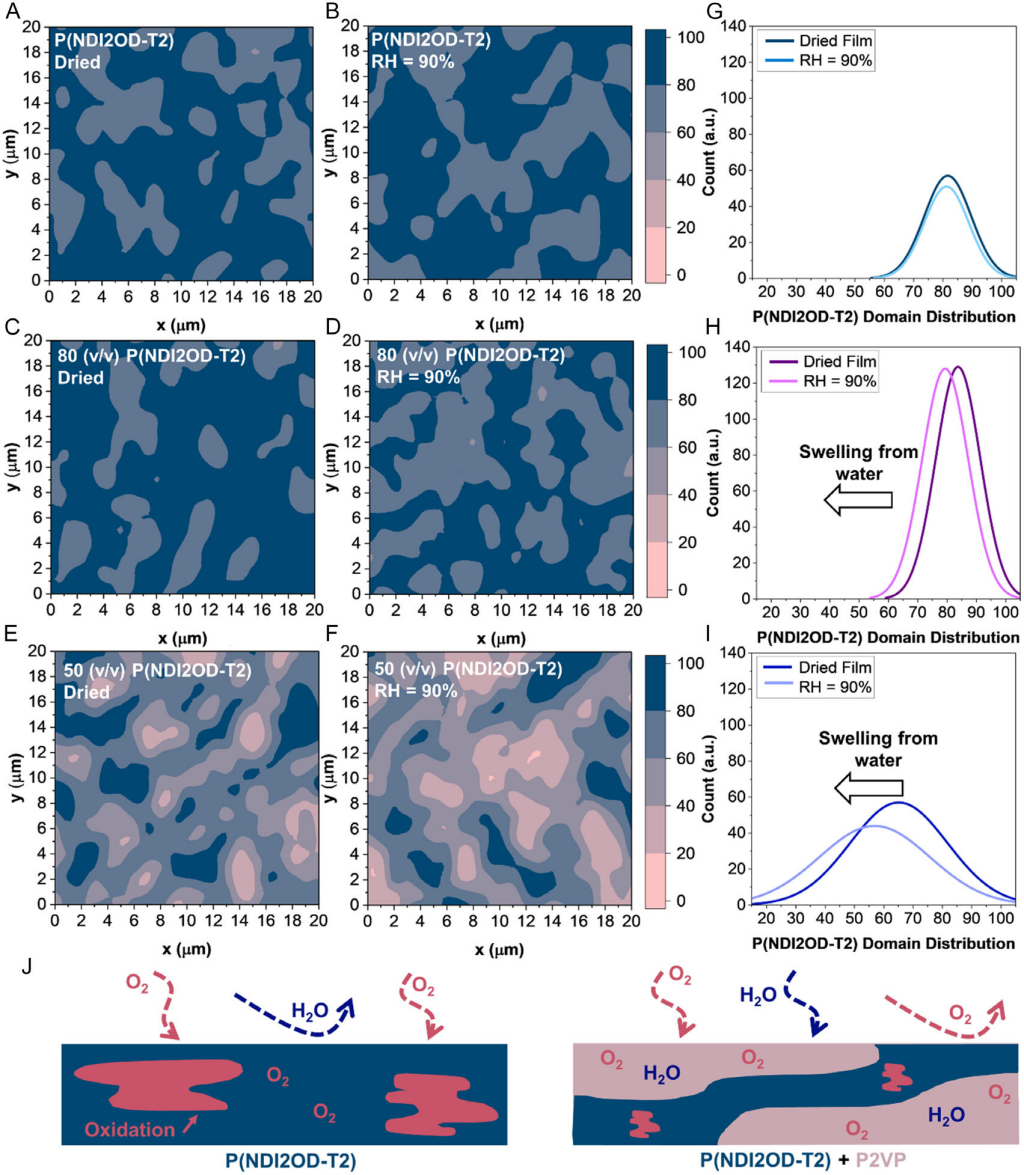
Relative P(NDI2OD‐T2) compositional distribution Raman maps for A,B) neat P(NDI2OD‐T2), C,D) 20/80, and E,F) 50/50 v/v P2VP/P(NDI2OD‐T2) blends, where P2VP has a molecular weight of 40 000 g mol^−1^. Films were either dried at 50 °C under vacuum or exposed to RH = 90% for 1 h. G–I) Corresponding histograms of P(NDI2OD‐T2) domain distributions derived from the Raman maps. J) Schematic of proposed stabilization mechanism, whereby atmospheric water absorbed by P2VP displaces oxygen from semiconductor domains and reducing oxidation within the film.

Raman maps were also used to illustrate the domain distribution of P(NDI2OD‐T2) and P2VP (Mn = 40 000 g mol^−1^) for films with blend ratios of 100, 80, and 50 vol% P(NDI2OD‐T2) to P2VP (**Figure** **5**, with supporting AFM scans in Figure S10, Supporting Information). As the content of P2VP increases, the size and purity of P(NDI2OD‐T2) domains decreases, correlating directly to initial OTFT performance (Figure S7, Supporting Information).^[^ ^22^, ^28^ ^]^ We hypothesize that films with higher P2VP content exhibit increased sensitivity to atmospheric moisture, resulting in a greater density of interface traps and more variable electrical performance. This is highlighted in Figure S9, Supporting Information, where both *V*_T_ and standard error of the blended films increase with P2VP content.

Raman maps and corresponding histograms also serve to highlight the water absorption process (Figure 5A–F), shown by films either dried under vacuum (50°C 1h) or exposed to RH = 90% for an hour. Neat P(NDI2OD‐T2) shows minimal change in semiconductor compositional distribution (Figure 5A,B,G). In contrast, blends containing 20% and 50% P2VP exhibit a clear downshift in domain purity and distribution upon exposure to humidity, suggesting that P2VP domains swell in the presence of water (Figure 5C–F,H–I). Interestingly, there appears to be a limit to observation on the micron scale. As films blended with 70% P2VP show only minor changes in semiconductor domain distribution when exposed to RH = 90% (Figure S11, Supporting Information), indicating that beyond a certain P2VP concentration, further swelling does not significantly influence P2VP domains.

Therefore, we surmise the stabilization observed in P(NDI2OD‐T2)/P2VP blends under ambient conditions arises from the hygroscopic properties of P2VP. We propose two plausible mechanistic pathways: 1) displacement of oxygen by water uptake into P2VP domains, and 2) oxygen partitioning into the hydrated P2VP regions (Figure  5J). As indicated by Raman and TGA, P2VP absorbs water, while neat P(NDI2OD‐T2) shows negligible hygroscopic properties. This absorbed water must occupy part of the film's free interstitial volume, reducing the probability of oxygen diffusion near the semicondctor.^[^ ^38^, ^59^, ^60^ ^]^ In addition to this, water‐rich P2VP domains may also serve as an oxygen sink, pulling the oxygen away from the semiconductor. Oxygen permeability in hydrophilic polymer networks, particularly hydrogels, are known to depend strongly on their water content, where increased permeability has been observed for increasing water uptake from the polymer network.^[^ ^59^, ^61^, ^62^, ^63^ ^]^ Therefore, oxygen may preferentially partition into the hydrated P2VP domains, reducing its concentration near the P(NDI2OD‐T2) domains.

While our results support both displacement and partitioning as plausible pathways, it is also possible that direct chemical interactions between the pyridine moieties of P2VP and oxygen also contribute to stabilization. In particular, pyridine groups may weakly complex or protonate under humid conditions, altering the local environment to reduce the reactivity of oxygen in the semiconductor.^[^ ^64^ ^]^ Further analysis of water uptake, oxygen transport, film morphology, and P2VP–oxygen interactions is needed to clarify their relative contributions. This mechanistic insight will establish how hygroscopic polymers influence degradation pathways in air‐sensitive organic semiconductors.

## 3 Conclusion

This study explores the mechanisms behind performance stabilization in insulator‐semiconductor OTFT blends. Specifically, blending the n‐type organic semiconductor P(NDI2OD‐T2) with high molecular weight P2VP in BGTC OTFTs improves device stability through changes in morphology driven by P2VP molecular weight. Higher molecular weight P2VP forms larger domains that appear to reduce oxidation of the semiconductor, thereby improving device operation over seven days.

Additionally, the role of humidity is examined, suggesting water absorbed by P2VP reduces oxidation of P(NDI2OD‐T2). Devices containing P2VP can be temporarily regenerated via mild thermal treatment, whereas devices without P2VP remain degraded. In the absence of P2VP, oxygen readily penetrates the film and oxidizes the semiconductor. These findings provide mechanistic insight into how hygroscopic insulator polymers alter oxygen–water interactions in blended films.

## 4 Experimental Section

4.1

4.1.1

##### Materials

The 2‐vinylpyridine (97%) and styrene (99%) were purchased from Sigma‐Aldrich (MO, USA) and were passed through a basic alumina column to remove radical inhibitors. N,N′‐dicyclohexylcarbodiimide (DCC, 99%) and N‐hydroxysuccinimide (NHS, 98%) was purchased from Oakwood Chemical. BlocBuilder (BB) was received from Arkema. Hexane and tetrahydrofuran (THF) were purchased from Caledon Chemical (Caledon, ON). P(NDI2OD‐T2) (OSO400, LOT#YY25180DC) was obtained from 1‐Materials. HPLC grade chloroform and trichloro(octyl)silane (OTS, 97%) was obtained from Sigma‐Aldrich. Both gold (99.99%) and chromium (99.99%) were obtained from Angstrom Engineering. All materials were used as received unless otherwise specified.

##### P2VP Synthesis

The initiator NHS‐BB was synthesized by coupling of BB‐MA and N‐hydroxysuccinimide following established literature (**ESI**).^[^ ^65^ ^]^ Poly(2‐vinylpyridine) with varying molecular weights were synthesized by NMP. The polymerizations were performed in a 25‐mL three‐neck round bottom flask fitted with a condenser and a magnetic stir bar. Two of the necks in the flask were sealed with glass stoppers while the center neck was connected to the condenser. The homopolymerization was done using bulk polymerization where NHS‐BB was dissolved in the 2VP monomer and, reagent ratios were selected to achieve target molecular weights (Table S1, Supporting Information). The solution was bubbled under nitrogen for 15 min and was heated to 120 °C under nitrogen for 3 h. The start of the reaction was taken when the target temperature was reached (*t* = 0 min). The reaction was then stopped, removed from heating, and precipitated three times into hexanes from tetrahydrofuran (THF). The final polymers were dried under vacuum overnight at room temperature. The different molecular weights were achieved by altering the amount of NHS‐BB added while keeping the amount of monomer consistent.

##### Gel Permeation Chromatography (GPC)

GPC of the P2VP homopolymers was performed using the multidetector Malvern OMNISEC GPC. It included an OMNISEC Resolve pump, an autosampler (CHR7100), two D6000M columns, an OMNISEC Reveal (CHR6000) differential refractive index, a diode‐array‐based UV/Vis spectrometer, and a Viscotek SEC‐MALS 20 multiangle light scattering detector. High‐performance liquid chromatography (HPLC) grade THF at 30 °C was used as the mobile phase (flow rate 1.0 mL min^−1^). A triple detection calibration was performed on the instrument using narrow molecular weight (Mw) polystyrene standards prepared by Malvern. Samples were dissolved in HPLC grade THF at 2.0 mg mL^−1^ concentration and filtered using a syringe filter with 0.22 μM PTFE membrane prior to analysis. The number average molecular weights (Mn) and dispersity's (Ð = Mw/Mn) were determined using the OMNISEC software. Average molecular weight is reported in Table S1, Supporting Information, while Figure S1a, Supporting Information, shows representative GPC curves for each molecular weight polymer.

##### Nuclear Magnetic Resonance Spectroscopy (NMR)

The polymerization conversion was monitored using NMR (Figure S1b, Supporting Information). For ^1^H‐NMR, a Bruker AVANCE II 400 MHz spectrometer was used. Samples were dissolved in deuterated chloroform (CDCl_3_).

##### Thin‐Film and Device Fabrication

Thin films were fabricated on prime silicon substrates (15 × 20 mm, WaferPro) with a 230 nm thermally grown silicon oxide dielectric diced by nanoFab (University of Alberta). Substrates were cleaned by sonication in sequential baths of soapy water (Contrex AL alkaline liquid detergent), distilled water, acetone, and methanol and then dried with nitrogen before being plasma treated for 10 min to remove residual solvents and roughen the substrate surface. Solutions of OTS were prepared at 1% v/v in anhydrous toluene in a controlled nitrogen filled glove box. Substrates were rinsed with distilled water and isopropanol then submerged in silane solutions for 1 h at 70 °C. Treated substrates were then rinsed with toluene and isopropanol and dried in a vacuum oven at 70 °C for 1 h.  P2VP and P(NDI2OD‐T2) solutions were prepared in chloroform at 20 mg mL^−1^, sealed with parafilm, and dissolved on a hot plate at 50 °C for an hour. P2VP/ P(NDI2OD‐T2) vol% solutions were prepared by adding the dissolved materials to a vial together in the appropriate ratios and left to sonicate for 90 min while wrapped in parafilm. The solutions were deposited by static spin coating at 2000 rpm for 90 s, followed by annealing at 150 °C for 1 h under vacuum. In the case of the neat P(NDI2OD‐T2) thin‐film, 20 mg mL^−1^ solution was used. The gate electrodes were deposited by physical vapor deposition using a shadow mask with a channel width of 1000 μm and channel length of 30 μm to create 20 individual BGTC transistors per substrate. A chromium interlayer that was deposited at a rate of 0.5 Å s^−1^ to a thickness of 20 Å, followed by the deposition of gold at a rate of 1 Å s^−1^ until a thickness of 500 Å was reached.

##### Device Characterization

Transistors were characterized using a custom‐built automatic multitester. The tester consists of 48 gold‐plated (20 nm) nickel probe tips, which contact the source‐drain electrodes of the individual transistors and the gate electrode and provide high throughput testing capability while introducing a resistance of ≈750 mOhm to testing.^[^ ^66^ ^]^ A Keithley 2614B and an MCC USB DAQ were used to control the source‐drain voltage (*V*_SD_) and gate voltage (*V*_GS_) to obtain source‐drain current (*I*_SD_) measurements. The multitester was kept at room temperature in air or in a N_2_ glovebox at atmospheric pressure for the duration of characterization. To obtain the transfer curves, the *V*_SD_ was fixed to a constant 50 V. The *V*_GS_ was swept from −10 to 50 V at a frequency of ≈10 Hz. Output curves were obtained by sweeping the *V*_SD_ from 0 to 50 V while holding the *V*_GS_ constant from 0 to 60 V.

To determine the saturation regime electron field‐effect mobility (*μ*_e_) the metal–oxide–semiconductor field‐effect transistor (MOSFET) equation was rearranged to isolate for *μ*_*e*_ (Equation (1)).^[^ ^25^ ^]^

(1) $$\mu_{e}=\frac{2L}{WC_{i}}{\left(\frac{\partial \sqrt{I_{\text{SD}}}}{\partial V_{\text{GS}}}\right)}^{2}$$

Here, *C*_i_ is the capacitance (F cm^−2^), and *W* (μm) and *L* (μm) are the channel width and length, respectively. Values presented are the average of the last four of five measurements from two substrates with 20 devices each, with error bars representing the standard deviation. Each device was operated at 50 V. Following the MOSFET equation, the threshold voltage (*V*_T_) was determined by plotting $\sqrt{I_{\text{SD}}}$ against $V_{\text{GS}}$, whereby the intersection of this curve with the $V_{\text{GS}}$‐axis gives *V*_T_.^[^ ^25^ ^]^ Transconductance (*g*_m_ *)* was calculated following Equation (2), taking the average transconductance in the saturation regime of the transfer curve.^[^ ^25^ ^]^ The on/off current ratio (*I*_ON/_ *I*_OFF_) was calculated using the minimum and maximum currents of the transfer curve.

(2) $$g_{m}={\left[\frac{\partial I_{\text{SD}}}{\partial V_{\text{GS}}}\right]}_{V_{\text{SD}}\;=\;\text{const}}$$

The trapped charge density at the dielectric/semiconductor interface ($N_{\text{int},\text{trap}}$) was determined following Equation 3, and the *SS* and corresponding interface defect density (*D*_int_) was computed by Equation 4 and 5, respectively.^[^ ^53^, ^67^ ^]^ Note here *q* is the electronic charge, *k* is Boltzmann constant, and *T* is the temperature.

(3) $$N_{\text{int},\text{trap}}=\frac{\left|V_{T}\right|C_{i}}{q}$$

(4) $$\text{SS}={\left(\frac{\partial \text{log}(I_{\text{SD)}}}{\partial V_{\text{GS}}}\right)}^{-1}$$

(5) $$D_{\text{int}}=\left(\frac{\text{SSq}}{\text{kTln}\left(10\right)}-1\right)\frac{C_{i}}{q}$$

Contact resistance of P(NDI2OD‐T2) has been previously studied and results in relatively negligible contact resistance for this device architecture and electrode composition.^[^ ^68^ ^]^

##### Raman Microscopy Maps

Raman maps were taken using a Renishaw inVia Qontor confocal Raman microscope equipped with a Leica Microsystems bright‐field microscope with a DM2700 light source. A 500 mW, 532 nm wavelength laser with a 2400 L mm^−1^ grating was used to obtain measurements in the spectral range of 900–2000 cm^−1^, focused on the sample by a X50L objective. Raman maps (20 × 20 μm) were generated from 400 individual spectra using a 1.0 μm step size with 5% laser power (25 mW) and a 1 s exposure time. To assess the amount of degradation from laser exposure, a sample image was taken before and after mapping (Figure S2, Supporting Information). Raman signals are baseline corrected using the WiRE software.

Raman compositional mapping was performed by plotting the intensity (normalized to thickness) of P(NDI2OD‐T2) C—C stretching peak, which occurs at 1610 cm^−1^, for each micron pixel on the Raman map.^[^ ^49^ ^]^ Each point of the map was normalized to the maximum intensity of this peak for a given map to observe how the overall signal varies across a given film (I/I_max_), providing a map of changes in the domain purity of P(NDI2OD‐T2) relative to its film. One 20 × 20 μm map was taken per film.

It is important to note that P2VP is inactive under 532 nm laser exposure, and thus there is no overlap between the C—C peaks of P2VP and P(NDI2OD‐T2) (Figure S3, Supporting Information). Moreover, it is active under a 785 nm wavelength laser, however, this wavelength causes P(NDI2OD‐T2) to fluoresce and saturate the detector (Figure S3, Supporting Information).^[^ ^69^ ^]^

##### Stability Characterization

Thin film electrical stability studies were conducted over a period of 10 days through August 2024 (RH of 40%–50%). On the first day, samples were left to sit for 30 min in air prior to being tested. Following this, samples are left in air for 3 days before being tested twice: once right away, and once after being heated at 55 °C for an hour under vacuum. The heating stage was used to remove any residual water absorbed from the atmosphere. This procedure was then repeated after the films had been left under atmosphere for 7 days. Films were left in air for 10 days total from the date of fabrication.

Raman humidity maps were performed as follows. Film samples were first heated at 50 °C for an hour to remove any moisture, and a map was measured. Following this, the films were exposed to the desired RH (90%) for an hour in the THMS600 Linkam stage controlled through the Linkam RHGen Humidity Controller.

##### Profilometry

Film thickness and roughness were computed by measuring and averaging six different measurements using a Dektak XT profilometer (Bruker).

##### AFM

AFM scans were taken using a Bruker Dimension FastScan AFM with ScanAsyst‐Air tips in PeakForce Tapping Mode. One or two scans were taken per sample. Imaging processing was performed with NanoScope Analysis v.3.0.

##### TGA

A Waters Discovery TGA 5500 was used to perform the TGA in 100 μL platinum pans. The unit was controlled by the TRIOS software package (v5.5.1). TGA measurements were conducted under nitrogen flow at 25 mL min^−1^. Samples were heated at a rate of 10 °C min^−1^ to 600 and 900 °C for P2VP and P(NDI2OD‐T2), respectively.

##### Modeling

Numerical simulations were used to investigate the device physics of OTFTs, using SILVACO's TCAD virtual device simulator software.^[^ ^55^, ^70^, ^71^, ^72^ ^]^ A 2D finite‐element numerical solver using Poisson's equation (Equation 6) and the drift‐diffusion model (Equation 7 and 8) was developed to apply to a 2D coordinate system that relates potential variation to space charge density.

(6) $$\text{div}\left(\varepsilon_{s}\nabla \varphi \right)=-\rho$$

Here, *φ* is the electrostatic potential, $\varepsilon_{s}$ is the semiconductor permittivity, and *ρ* is the space charge density.

(7) $${\vec{J}}_{n}=qn\mu_{n}\vec{E}+qD_{n}\nabla n$$

(8) $${\vec{J}}_{p}=qn\mu_{p}\vec{E}-qD_{p}\nabla p$$

Here, respectively, ${\vec{J}}_{n}$ and ${\vec{J}}_{p}$ are the electron and hole density vectors, $\vec{E}$ is the electric field vector, $\mu_{n}$ and $\mu_{p}$ are the electron and hole mobility, $D_{n}$ and $D_{p}$ are the electron and hole diffusion coefficients, *q* is the elementary charge, and *n* and *p* are the electron and hole concentrations. In this work, we selectively changed the fixed interface charge density (*N*_int,fixed_) at the semiconductor/insulator interface while fixing all the other parameters to investigate the threshold voltage shift observed in experimental transfer characteristics of the OTFTs.

##### GIWAXS

GIWAXS experiments were performed at the SOLEIL Synchrotron facility in Saint‐Aubin, France using the SIRIUS beamline (10 keV). GIWAXS measurements were taken directly from the polymer samples deposited on silicon substrates. A photon energy was selected using a Si(111) monochromator and the angle of incidence set according to the sample, approximately in the range of 0.15°. GIWAXS patterns were collected with a Rayonix MX300 CCD detector (73 × 73 μm pixel size), which was placed ≈312 mm from the sample center. Five scans are summed and averaged. The GIWAXS data was then calibrated against a silver behenate and P3HT standard and analyzed using the GIXSGUI software package.^[^ ^73^ ^]^

##### Statistical Analysis

Averages and standard deviations reported in this work were typically calculated from two chips with 20 devices each (total of 40 devices) per condition. Analysis was performed using OriginLab, with specific data treatment and sample sizes described in their respective Experimental Section's Subsection.

## Supporting Information

Supporting Information is available from the Wiley Online Library or from the author.

## Conflict of Interest

The authors declare no conflict of interest.

## Supporting information

Supplementary Material

## Data Availability

The data that support the findings of this study are available from the corresponding author upon reasonable request.

## References

[1] Y. Wen , Y. Liu , Adv. Mater. 2010, 22, 1331. 20437478 10.1002/adma.200901454

[2] O. A. Melville , B. H. Lessard , T. P. Bender , ACS Appl. Mater. Interfaces 2015, 7, 13105. 26000612 10.1021/acsami.5b01718

[3] S. R. Forrest , in Organic Electronics: Foundations to Applications, Oxford University Press 2020, pp. 803–917.

[4] Y. Sui , Y. Deng , T. Du , Y. Shi , Y. Geng , Mater. Chem. Front. 2019, 3, 1932.

[5] Z. Zhang , J. K. W. Ho , C. Zhang , H. Yin , Z. Wen , G. Cai , R. Zhao , R. Shi , X. Lu , J. Liu , X. Hao , C. Cheng , S. K. So , J. Mater. Chem. C Mater. 2021, 9, 12281.

[6] S. Brixi , C. Dindault , B. King , H. R. Lamontagne , A. J. Shuhendler , S. Swaraj , B. H. Lessard , Adv. Electron. Mater. 2024, 10, 2300660.

[7] S. Griggs , A. Marks , H. Bristow , I. McCulloch , J. Mater. Chem. C Mater. 2021, 9, 8099. 34277009 10.1039/d1tc02048j PMC8264852

[8] S. Brixi , O. A. Melville , B. Mirka , Y. He , A. D. Hendsbee , H. Meng , Y. Li , B. H. Lessard , Sci. Rep. 2020, 10, 4014. 32132588 10.1038/s41598-020-60812-x PMC7055259

[9] Z. Genene , W. Mammo , E. Wang , M. R. Andersson , Adv. Mater. 2019, 31, 1807275. 10.1002/adma.201807275 30790384

[10] A. F. Paterson , S. Singh , K. J. Fallon , T. Hodsden , Y. Han , B. C. Schroeder , H. Bronstein , M. Heeney , I. McCulloch , T. D. Anthopoulos , Adv. Mater. 2018, 30, 1801079. 10.1002/adma.201801079 30022536

[11] Y. Wang , T. Michinobu , J. Mater. Chem. C Mater. 2018, 6, 10390.

[12] L. Shi , Y. Guo , W. Hu , Y. Liu , Mater. Chem. Front. 2017, 1, 2423.

[13] J. Dong , Y. Wang , T. Mori , T. Michinobu , Jpn. J. Appl. Phys. 2020, 59, SDDC05.

[14] J. Zaumseil , H. Sirringhaus , Chem. Rev. 2007, 107, 1296. 17378616 10.1021/cr0501543

[15] Z. Ding , G. Abbas , H. E. Assender , J. J. Morrison , S. G. Yeates , E. R. Patchett , D. M. Taylor , ACS Appl. Mater. Interfaces 2014, 6, 15224. 25116597 10.1021/am503560d

[16] C.‐S. Chuang , F.‐C. Chen , H.‐P. D. Shieh , Org. Electron. 2007, 8, 767.

[17] Y. Zhao , G. Dong , L. Wang , Y. Qiu , Appl. Phys. Lett. 2007, 90, 252110.

[18] N. Wrachien , A. Cester , D. Bari , J. Kovac , J. Jakabovic , M. Weis , D. Donoval , G. Meneghesso , IEEE Trans. Nucl. Sci. 2012, 59, 2979.

[19] Y. Li , Y. Xiong , H. Yang , K. Cao , R. Chen , J. Mater. Res. 2020, 35, 681.

[20] D. Radic , M. Stanojevic , M. Obradovic , A. Jovovic , Therm. Sci. 2017, 21, 1067.

[21] J. D. Núñez , A. M. Benito , S. Rouzière , P. Launois , R. Arenal , P. M. Ajayan , W. K. Maser , Chem. Sci. 2017, 8, 4987. 28989597 10.1039/c7sc00223h PMC5625303

[22] A. Gumyusenge , D. T. Tran , X. Luo , G. M. Pitch , Y. Zhao , K. A. Jenkins , T. J. Dunn , A. L. Ayzner , B. M. Savoie , J. Mei , Science 2018, 362, 1131. 30523104 10.1126/science.aau0759

[23] W. W. McNutt , A. Gumyusenge , L. A. Galuska , Z. Qian , J. He , X. Gu , J. Mei , ACS Appl. Polym. Mater. 2020, 2, 2644.

[24] J. E. Anthony , A. Facchetti , M. Heeney , S. R. Marder , X. Zhan , Adv. Mater. 2010, 22, 3876. 20715063 10.1002/adma.200903628

[25] W. Boukhili , S. Wageh , X. Wan , Z. Yu , C. L. Tan , H. Sun , Y.‐Y. Noh , K.‐J. Baeg , Y. Xu , D. Khim , Org. Electron. 2025, 138, 107191.

[26] S. Goffri , C. Müller , N. Stingelin‐Stutzmann , D. W. Breiby , C. P. Radano , J. W. Andreasen , R. Thompson , R. A. J. Janssen , M. M. Nielsen , P. Smith , H. Sirringhaus , Nat. Mater. 2006, 5, 950. 17128260 10.1038/nmat1779

[27] H. Jia , T. Lei , J. Mater. Chem. C Mater. 2019, 7, 12809.

[28] S. Wang , S. Fabiano , S. Himmelberger , S. Puzinas , X. Crispin , A. Salleo , M. Berggren , Proc. Natl. Acad. Sci. 2015, 112, 10599. 26261305 10.1073/pnas.1501381112 PMC4553794

[29] X. Zhang , B. Wang , L. Huang , W. Huang , Z. Wang , W. Zhu , Y. Chen , Y. Mao , A. Facchetti , T. J. Marks , Sci. Adv. 2020, 6, eaaz1042. 32232157 10.1126/sciadv.aaz1042 PMC7096165

[30] S. Riera‐Galindo , F. Leonardi , R. Pfattner , M. Mas‐Torrent , Adv. Mater. Technol. 2019, 4, 1900104.

[31] A. D. Scaccabarozzi , N. Stingelin , J. Mater. Chem. A 2014, 2, 10818.

[32] C. E. Cunin , R. F. Meacham , E. R. Lee , H. Roh , S. Samal , W. Li , J. R. Matthews , Y. Zhao , M. He , A. Gumyusenge , ACS Appl. Mater. Interfaces 2024, 16, 39717. 39036945 10.1021/acsami.4c06609

[33] R. Noriega , J. Rivnay , K. Vandewal , F. P. V. Koch , N. Stingelin , P. Smith , M. F. Toney , A. Salleo , Nat. Mater. 2013, 12, 1038. 23913173 10.1038/nmat3722

[34] J. G. Kennemur , Macromolecules 2019, 52, 1354.

[35] Z. Wang , K. Wang , C. Eom , Y. Chen , G. Sun , M. Kim , J. M. Montes de Oca , D. Liang , K. Bagchi , S. N. Patel , J. J. de Pablo , P. F. Nealey , Adv. Funct. Mater. 2025, e14589.

[36] A. Noro , Y. Tomita , Y. Shinohara , Y. Sageshima , J. J. Walish , Y. Matsushita , E. L. Thomas , Macromolecules 2014, 47, 4103.

[37] S. A. Shamsudin , T. Mikihito , H. Hirokazu , Macromol. Symp. 2017, 371, 75.

[38] E. S. Cho , C. M. Evans , E. C. Davidson , M. L. Hoarfrost , M. A. Modestino , R. A. Segalman , J. J. Urban , ACS Macro Lett. 2015, 4, 70. 35596375 10.1021/mz500765y

[39] B. A. Junisu , I. Ching‐Ya Chang , Y.‐S. Sun , Langmuir 2022, 38, 13009. 36263886 10.1021/acs.langmuir.2c01173

[40] J. Teng , S. Bates , D. A. Engers , K. Leach , P. Schields , Y. Yang , J. Pharm. Sci. 2010, 99, 3815. 20665845 10.1002/jps.22204

[41] H. R. Lamontagne , B. H. Lessard , ACS Appl. Polym. Mater. 2020, 2, 5327.

[42] T. Steckmann , I. Angunawela , S. Kashani , Y. Zhu , M. M. Nahid , H. Ade , A. Gadisa , Adv. Electron. Mater. 2022, 8, 2101324.

[43] H. Yan , Z. Chen , Y. Zheng , C. Newman , J. R. Quinn , F. Dötz , M. Kastler , A. Facchetti , Nature 2009, 457, 679. 19158674 10.1038/nature07727

[44] M. Ali , R. B. Ewenike , J. G. Manion , B. H. Lessard , ACS Appl. Mater. Interfaces 2025, 17, 1734. 39706816 10.1021/acsami.4c17317

[45] X. Rodríguez‐Martínez , M. S. Vezie , X. Shi , I. McCulloch , J. Nelson , A. R. Goñi , M. Campoy‐Quiles , J. Mater. Chem. C Mater. 2017, 5, 7270.

[46] L. Xue , W. Li , G. G. Hoffmann , J. G. P. Goossens , J. Loos , G. de With , Macromolecules 2011, 44, 2852.

[47] S. K. Schoustra , M. H. P. de Heer Kloots , J. Posthuma , D. van Doorn , J. A. Dijksman , M. M. J. Smulders , Macromolecules 2022, 55, 10341. 36530523 10.1021/acs.macromol.2c01595 PMC9753755

[48] M. J. Dyson , E. Lariou , J. Martin , R. Li , H. Erothu , G. Wantz , P. D. Topham , O. J. Dautel , S. C. Hayes , P. N. Stavrinou , N. Stingelin , Chem. Mater. 2019, 31, 6540.

[49] I. Denti , S. Cimò , L. Brambilla , A. Milani , C. Bertarelli , M. Tommasini , C. Castiglioni , Chem. Mater. 2019, 31, 6726.

[50] E. Giussani , D. Fazzi , L. Brambilla , M. Caironi , C. Castiglioni , Macromolecules 2013, 46, 2658.

[51] R. Bishnu , R. Mukherjee , N. Bhandaru , A. Dutta , Soft Matter 2025, 21, 5284. 40485499 10.1039/d5sm00335k

[52] Q. Wang , F. Cui , M. Sha , C. Zhang , X. Liao , H. Yin , X. Hao , Adv. Funct. Mater. 2023, 33, 2304169.

[53] W. Boukhili , M. Mahdouani , M. Erouel , J. Puigdollers , R. Bourguiga , Synth. Met. 2015, 199, 303.

[54] Y. E. Kim , H. Jung , J. H. Park , H. Yoo , C.‐H. Kim , Org. Electron. 2025, 138, 107195.

[55] H. Lee , Y. E. Kim , J. Bae , S. Jung , R. A. Sporea , C.‐H. Kim , ACS Appl. Mater. Interfaces 2023, 15, 10918. 36799771 10.1021/acsami.2c22350

[56] S.‐W. Jo , J. Choi , R. Hayakawa , Y. Wakayama , S. Jung , C.‐H. Kim , J. Mater. Chem. C Mater. 2021, 9, 15415.

[57] M. Nikolka , G. Schweicher , J. Armitage , I. Nasrallah , C. Jellett , Z. Guo , M. Hurhangee , A. Sadhanala , I. McCulloch , C. B. Nielsen , H. Sirringhaus , Adv. Mater. 2018, 30, 1801874. 10.1002/adma.201801874 30022541

[58] M. Nikolka , I. Nasrallah , B. Rose , M. K. Ravva , K. Broch , A. Sadhanala , D. Harkin , J. Charmet , M. Hurhangee , A. Brown , S. Illig , P. Too , J. Jongman , I. McCulloch , J.‐L. Bredas , H. Sirringhaus , Nat. Mater. 2017, 16, 356. 27941806 10.1038/nmat4785

[59] L. Ansaloni , M. Minelli , M. Giacinti Baschetti , G. C. Sarti , J. Membr. Sci. 2014, 471, 392.

[60] B. Bugbee , M. Blonquist , Absolute and Relative Gas Concentration: Understanding Oxygen in Air, Apogee Instruments.

[61] M. F. Refojo , F.‐L. Leong , J. Membr. Sci. 1978, 4, 415.

[62] A. Okugawa , Y. Yuguchi , D. Hayakawa , F. Ueno , K. Hatai , C. Yamane , Carbohydr Polym 2023, 313, 120849. 37182949 10.1016/j.carbpol.2023.120849

[63] K. K. Mokwena , J. Tang , M.‐P. Laborie , J. Food Eng. 2011, 105, 436.

[64] M. C. Sicilia , A. Niño , C. Muñoz‐Caro , J. Phys. Chem. A 2005, 109, 8341. 16834225 10.1021/jp050530n

[65] J. Vinas , N. Chagneux , D. Gigmes , T. Trimaille , A. Favier , D. Bertin , Polymer 2008, 49, 3639.

[66] J. Manion , B. H. Lessard , Nat. Rev. Mater. 2024, 9, 377.

[67] Z. J. Comeau , R. R. Cranston , H. R. Lamontagne , C. S. Harris , A. J. Shuhendler , B. H. Lessard , Commun Chem 2022, 5, 178. 36697684 10.1038/s42004-022-00797-y PMC9814745

[68] N. J. Dallaire , S. Brixi , M. Claus , S. Blawid , B. H. Lessard , Appl. Phys. Rev. 2022, 9, 011418.

[69] G. Wen , X. Zou , R. Hu , J. Peng , Z. Chen , X. He , G. Dong , W. Zhang , RSC Adv. 2021, 11, 20191. 35479889 10.1039/d1ra01474a PMC9033976

[70] J. Bae , S. Park , H. Jung , E.‐H. Ko , I. Kymissis , C.‐H. Kim , J. Mater. Sci.: Mater. Electron. 2024, 35, 185.

[71] S.‐W. Jo , S. Cho , C.‐H. Kim , J. Phys. D: Appl. Phys. 2022, 55, 405101.

[72] M. Ali , B. Ronnasi , M. Ourabi , J. H. Park , J.‐P. St‐Pierre , C.‐H. Kim , B. H. Lessard , Mater. Adv. 2025, 6, 557.

[73] Z. Jiang , J. Appl. Crystallogr. 2015, 48, 917.

