# Supplementary material for: Tuning the Air Stability of N‐Type Semiconductors via Poly(2‐vinylpyridine): The Importance of Humidity and Molecular Weight
Source: Small Sci. 2025 Oct 23;5(12):e202500452. doi: 10.1002/smsc.202500452 (PMC12697774; doi:10.1002/smsc.202500452)
Supplement: Supplementary file 1 — Supplementary Material [file SMSC-5-e202500452-s001.pdf]

Tuning the Air Stability of N-Type Semiconductors via Poly(2-vinylpyridine): the Importance of Humidity and Molecular Weight.

Supporting Information

Laura E. Dickson[1], Vittoria-Ann Dipalo[1], Trevor Plint[1], Kannan Udaya Mohanan[2], Joseph G. Manion[1], Chang-Hyun Kim[2], and Benoît H. Lessard[1,2]

1. Department of Chemical and Biological Engineering, University of Ottawa, 161 Louis Pasteur, Ottawa, Ontario K1N 6N5, Canada.
2. School of Electrical Engineering and Computer Science, University of Ottawa, 800 King Edward Ave. Ottawa, Ontario K1N 6N5, Canada.

*Corresponding author: benoit.lessard@uottawa.ca

1. Synthesis of P2VP Initiator.

Preparation of NHS-BlocBuilder. THF was treated with 3Å molecular sieve prior to use. Blocbuilder (1.000 g, 2.62 mmol, 1.0 equiv.) and NHS (0.362 g, 3.14 mmol, 1.2 equiv.) were dissolved in THF (5.0 mL), then the reaction solution was cooled with an ice bath for 30 min. While cooled, DCC (0.920 g, 4.46 mmol, 1.7 equiv.) was added to the reaction solution. The reaction was stirred while cooled with an ice bath for 1.5 hrs. The crude product solution was vacuum filtered, the retentate was rinsed with an additional amount of THF (20 mL), then the flow through was collected. The excess solvent was rotovapped, then the product was dried in vacuo at room temperature. The crude product was used without further purification to produce a white, beige solid (1.930g).

2. Additional Tables.

Table S1. Synthesis reagent and molecular weight of P2VP library.

Polymer	Amount of NHS-BlocBuilder Added	$m_{monomer}$ (g)	Conversion (%)	PDI	Actual Mn (kg/mol)
P2VP (5000 g/mol)	0.133g	2.92	67	1.6	5.97
P2VP (15000 g/mol)	0.022g	2.92	24	1.3	15.24
P2VP (40000 g/mol)	0.022g	2.92	62	2.3	38.95

Table S2. Semiconductor layer thickness and roughness determined by profilometry. Averages are presented plus/minus the standard deviation of 4 individual measurements.

Sample	Thickness (A)	Roughness (A)
**Ratio for MW 40 000 P2VP**		
20/80	3630.09 ± 201.9	66.9 ± 38.6
50/50	2718.0 ± 274.3	127.5 ± 20.8
70/30	2184.3 ± 42.6	224.9 ± 11.4
**Different MW 50/50 Ratio**		
15 000 g/mol	2277.8 ±26.2	107.2 ± 58.8
6000 g/mol	2370.8 ± 120.0	256.0 ± 56.0
40 000 g/mol	2718.0 ± 274.3	127.5 ± 20.8
**P(NDI2OD-T2)**		
P(NDI2OD-T2)	3257.5 ± 400.6	43.7 ± =2.4

Table S3. Summary of experimental electrical parameters for 20/80 P2VP/P(NDI2OD-T2) blend devices on day 1 and day 3 before and after drying at 50ºC in vacuum for 1 hour. Results are presented as an average of 40 transistor devices.

Parameter	Unit	P2VP/P(NDI2OD-T2)					
		Day 1		Day 3		Day 3, dried	
		Avg	std	Avg	std	Avg	std
Mobility	$cm^2 V^{-1} s^{-1}$	1.43E-03	5.20E-04	1.02E-04	3.12E-05	3.70E-04	1.98E-04
I_{ON}	A	9.54E-07	2.43E-07	3.15E-08	7.87E-09	1.30E-07	6.00E-08
Ioff	A	3.51E-09	3.18E-09	2.17E-11	9.75E-12	1.91E-11	1.12E-11
I_{OFF}	cm^{-2}	5.83E+11	2.11E+11	2.63E+12	3.38E+11	1.80E+12	3.47E+11
VT	V	-4.35E+00	3.23E+00	2.83E+01	1.13E+03	1.67E+01	5.75E+00
I_{ON}/I_{OFF}	-	8.17E+02	-	1.88E+03	-	9.83E+03	-
D_{int}	$cm^{-2} eV^{-1}$	3.09E+13	8.74E+12	6.37E+12	1.77E+12	3.06E+12	7.95E+11
SS	V/dec	1.96E+01	5.52E+00	4.08E+00	1.12E+00	2.09E+00	5.52E-01
N_{fixed}	cm^{-2}	-9.00E+11	-	-2.80E+12	-	-4.00E+11	-

Table S4. Summary of experimental electrical parameters for neat P(NDI2OD-T2) devices on day 1 and day 3 before and after drying at 50ºC in vacuum for 1 hour. Results are presented as an average of 40 transistor devices.

Parameter	Unit	P(NDI2OD-T2)					
		Day 1		Day 3		Day 3, dried	
		Avg	std	Avg	std	Avg	std
Mobility	$cm^2 V^{-1} s^{-1}$	6.53E-04	3.23E-04	-	-	2.62E-04	2.84E-04
I_{ON}	A	9.39E-08	2.67E-08	-	-	1.96E-08	1.09E-08
Ioff	A	9.49E-12	4.02E-12	-	-	2.77E-11	6.78E-12
I_{OFF}	cm^{-2}	3.17E+12	1.39E+11	-	-	4.44E+12	4.01E+11
VT	V	3.72E+01	4.83E+00	-	-	4.91E+01	1.73E+01
I_{ON}/I_{OFF}	-	1.18E+04	-	-	-	5.63E+02	-
D_{int}	$cm^{-2} eV^{-1}$	4.55E+12	1.69E+12	-	-	4.76E+12	7.85E+11
SS	V/dec	2.93E+00	1.07E+00	-	-	3.06E+00	4.95E-01
N_{fixed}	cm^{-2}	-2.90E+12	-	-	-	-4.60E+12	-

3. Additional Figures.

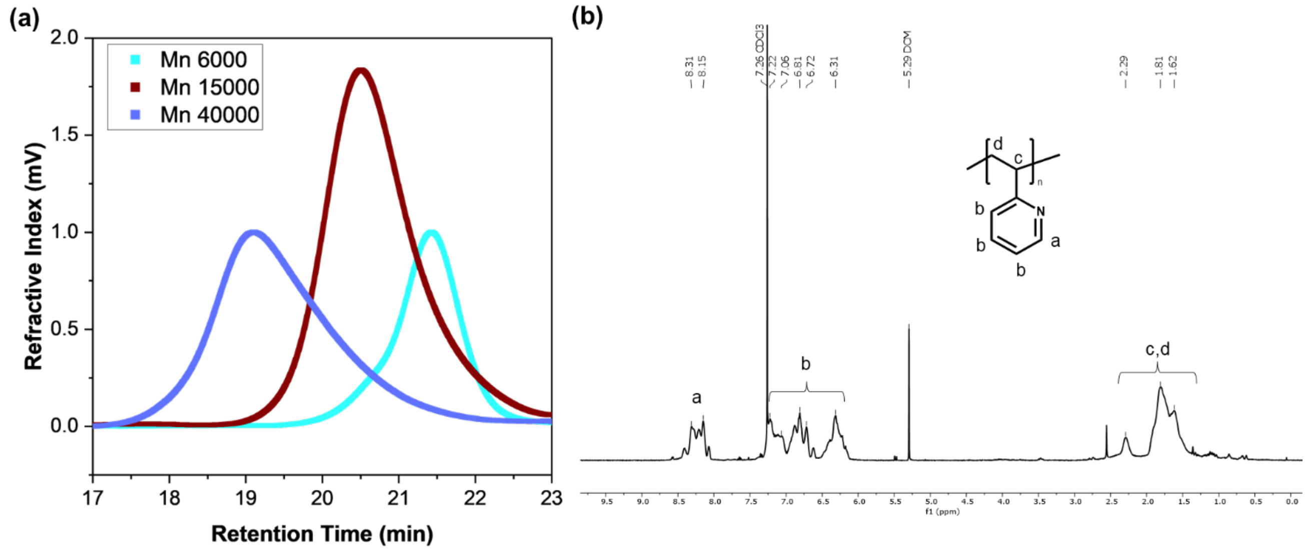

Figure S1. (a) Gel permeation chromatograms of P2VP with varying molecular weights synthesized by NMP. **(b)** Representative ^{1}H NMR spectra of the P2VP polymer series in $CDCl_3$. The spectra shown here represents the NMR taken for the 15000 g/mol P2VP polymer sample.

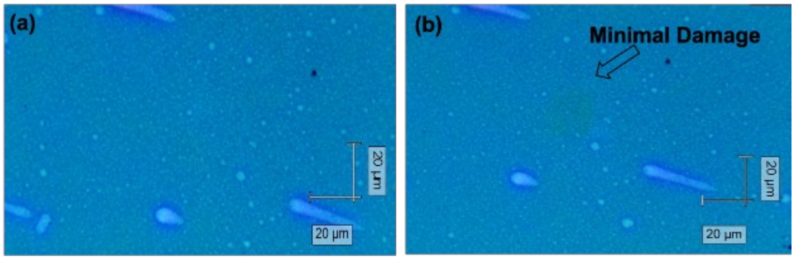

Figure S2. Sample microscopy image with a x50L magnifier for the sample map area **(a)** before and **(b)** after polarized maps were taken for films made with 50/50 40000 g/mol P2VP/ P(NDI2OD-T2).

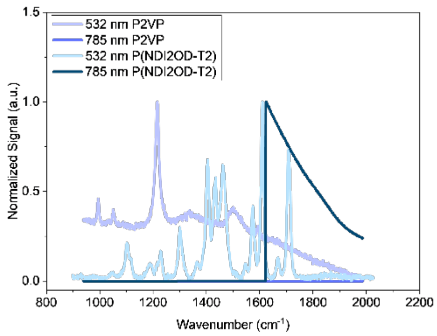

Figure S3. Raman of P2VP and P(NDI2OD-T2) under 532 and 785 nm wavelength.

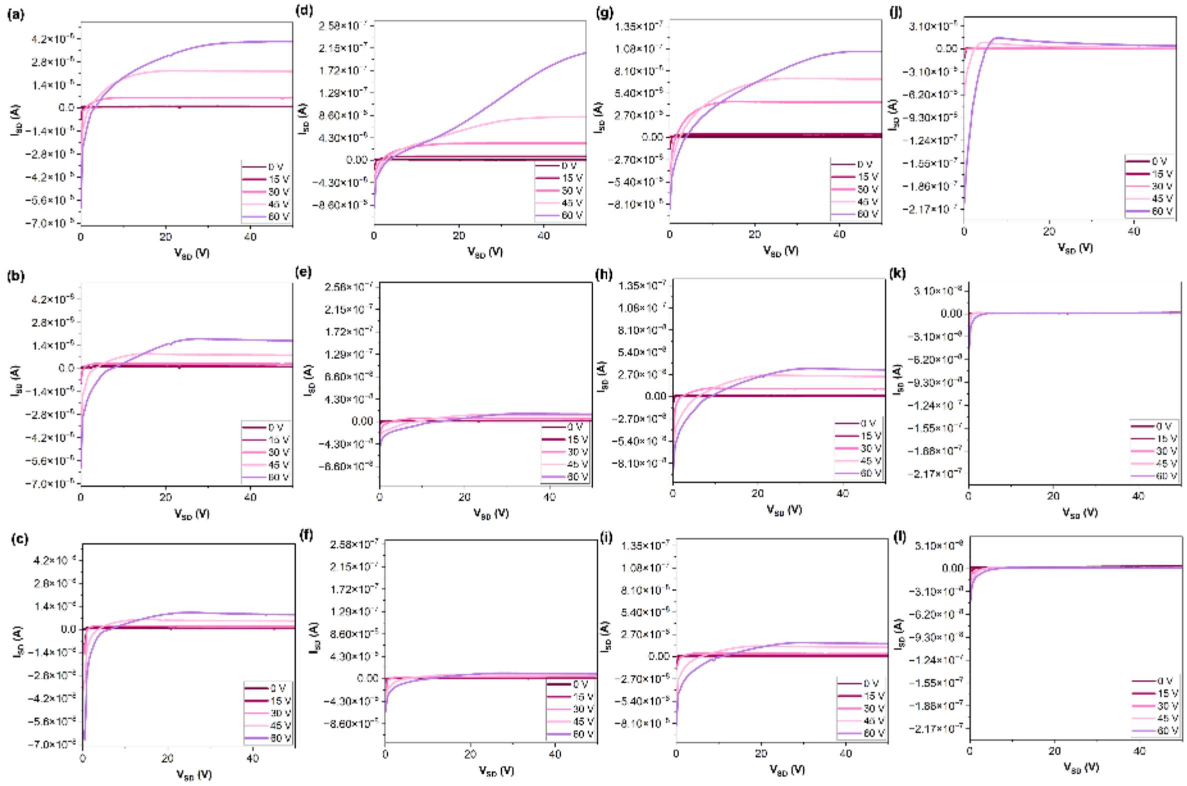

Figure S4. Sample output curves for OTFT devices fabricated with a volume ratio of 50/50 P2VP/P(NDI2OD-T2) for day 1 (top), day 3 (middle), and day 7 (bottom) for P2VP with a molecular weight of **(a-c)** 6000 g/mol, **(d-f)** 15000 g/mol, **(g-i)** 40000 g/mol, and **(j-l)** neat P(NDI2OD-T2).

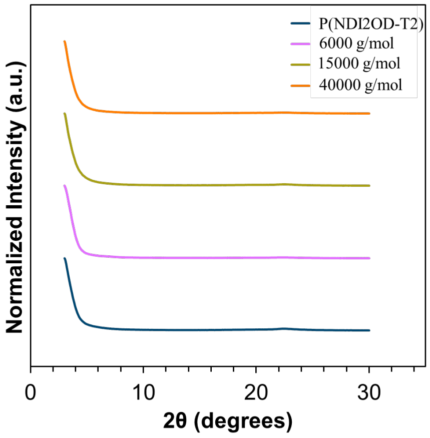

Figure S5. XRD pattern for various molecular weight 50/50 P2VP/ P(NDI2OD-T2) samples.

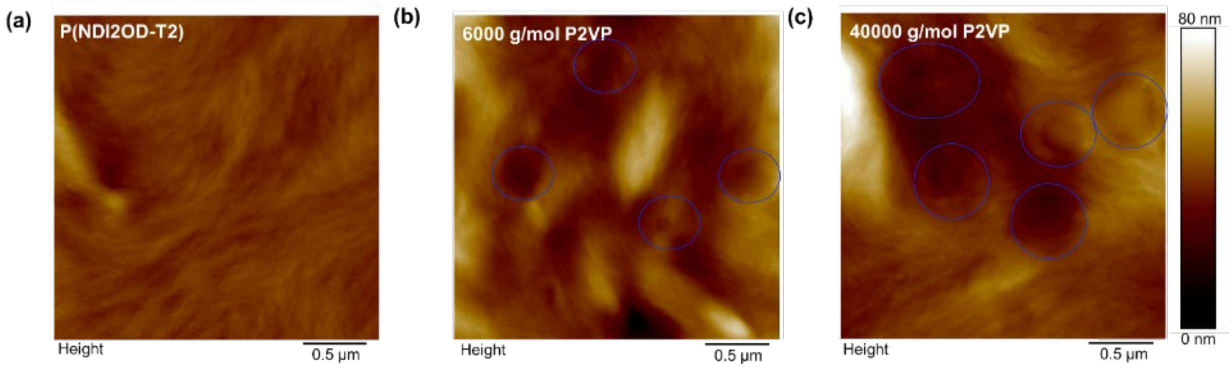

Figure S6. Atomic force microscopy with highlighted features for films fabricated with **(a)** neat P(NDI2OD-T2) or a 50/50 blend ratio of P(NDI2OD-T2)/P2VP with a M_n of **(b)** 6000 g/mol and **(c)** 40000 g/mol. Note all three maps share the scale bar in **(c)**.

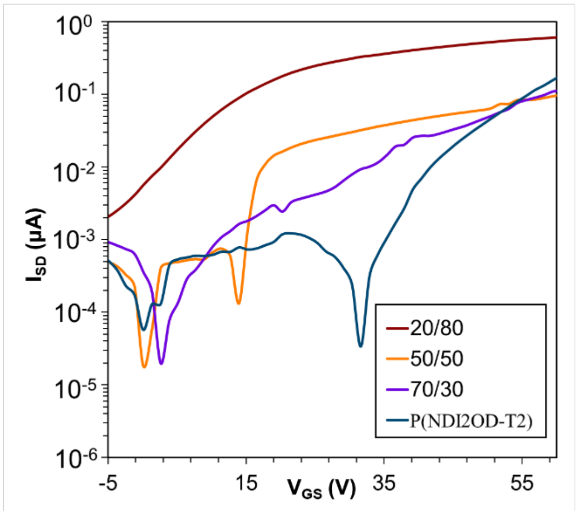

Figure S7. Sample transfer curves for OTFT devices fabricated with varying volume ratios of P2VP/P(NDI2OD-T2) and a P2VP of 40000 g/mol.

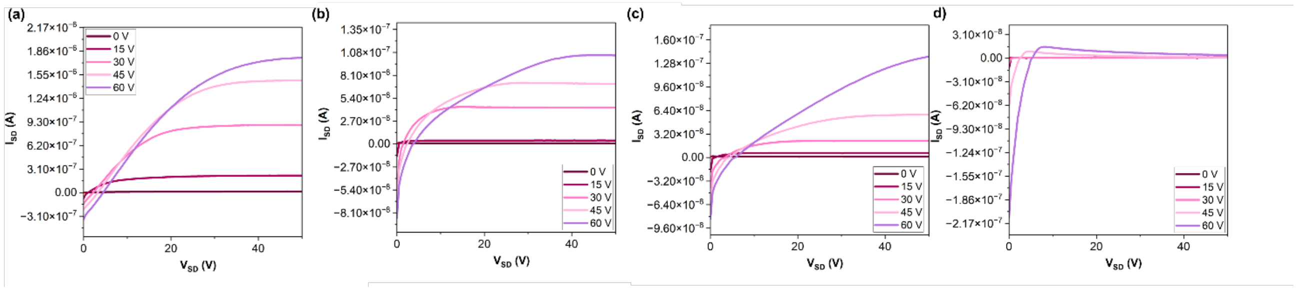

Figure S8. Sample output curves for OTFT devices fabricated with varying volume ratios of P2VP/P(NDI2OD-T2) and a molecular weight of P2VP of 40000 g/mol, where **(a)** 20/80, **(b)** 50/50, **(c)** 70/30, **(d)** neat P(NDI2OD-T2).

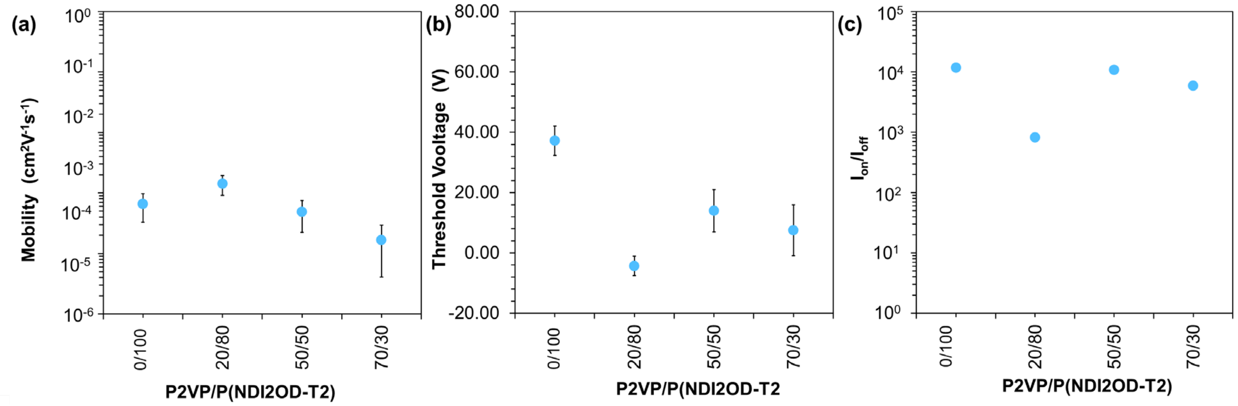

Figure S9. For OTFTs fabricated with varying volume ratios of P2VP/P(NDI2OD-T2) and a P2VP of 40000 g/mol, the average **(a)** electron mobility (μ_e), **(b)** threshold voltage (V_T), **(c)** I_{ON}/I_{OFF}. Values presented are the average of the last four of five measurements from 40 devices, with error bars representing the standard deviation. Each device was operated at 50 V_{SD}. Averages are presented from 40 OTFT devices.

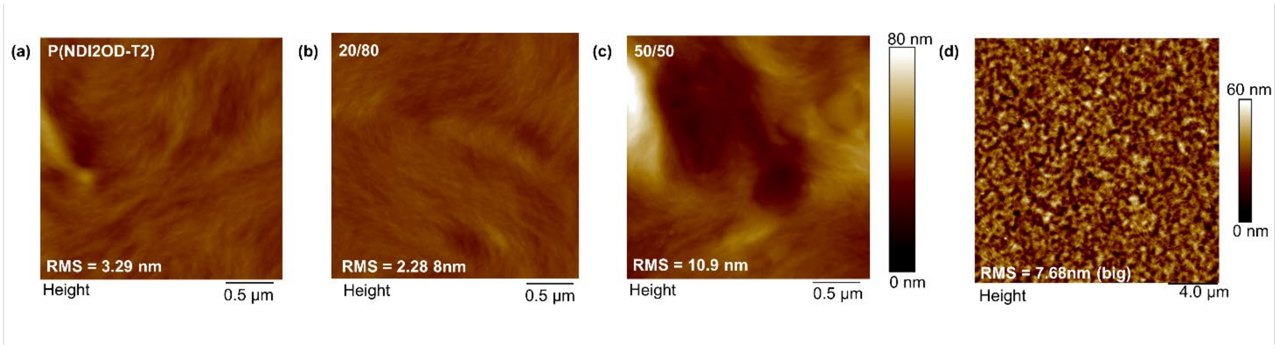

Figure S10. 2.5 x 2.5 µm AFM scans for **(a)** neat and P2VP/ P(NDI2OD-T2) volume blends with ratios of **(b)** 20/80 and **(c)** 50/50. **(d)** 20 x 20 µm AFM scan for the 20/80 semiconductor-insulator blend.

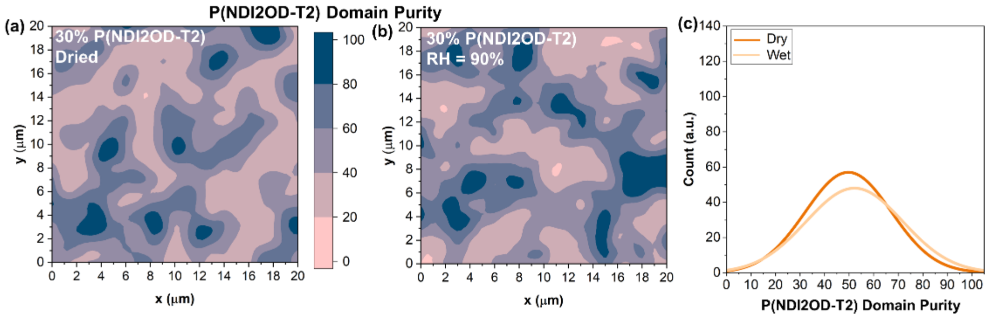

Figure S11. Relative P(NDI2OD-T2) domain distribution Raman maps for a film fabricated with 70/30 P2VP/P(NDI2OD-T2) when **(a)** dried for 1 hour at 50°C under vacuum and **(b)** exposed to RH = 90% for 1 hour. **(c)** Histograms of the distribution of P(NDI2OD-T2) domains.
